# Programming of Embryonic Development

**DOI:** 10.3390/ijms222111668

**Published:** 2021-10-28

**Authors:** Carl R. Dahlen, Pawel P. Borowicz, Alison K. Ward, Joel S. Caton, Marta Czernik, Luca Palazzese, Pasqualino Loi, Lawrence P. Reynolds

**Affiliations:** 1 Center for Nutrition and Pregnancy, Department of Animal Sciences, North Dakota State University, Fargo, ND 58108, USA; carl.dahlen@ndsu.edu (C.R.D.); pawel.borowicz@ndsu.edu (P.P.B.); alison.ward@ndsu.edu (A.K.W.); joel.caton@ndsu.edu (J.S.C.); 2 Faculty of Veterinary Medicine, University of Teramo, 64100 Teramo, Italy; mczernik@unite.it (M.C.); ploi@unite.it (P.L.); 3 Institute of Genetics and Animal Biotechnology of the Polish Academy of Sciences, Warsaw, Jastrzębiec, 05-552 Magdalenka, Poland； lpalazzese@unite.it

**Keywords:** maternal nutrition, assisted reproductive techniques, developmental programming, epigenetics, early pregnancy, reproductive function

## Abstract

Assisted reproductive techniques (ART) and parental nutritional status have profound effects on embryonic/fetal and placental development, which are probably mediated via “programming” of gene expression, as reflected by changes in their epigenetic landscape. Such epigenetic changes may underlie programming of growth, development, and function of fetal organs later in pregnancy and the offspring postnatally, and potentially lead to long-term changes in organ structure and function in the offspring as adults. This latter concept has been termed developmental origins of health and disease (DOHaD), or simply developmental programming, which has emerged as a major health issue in animals and humans because it is associated with an increased risk of non-communicable diseases in the offspring, including metabolic, behavioral, and reproductive dysfunction. In this review, we will briefly introduce the concept of developmental programming and its relationship to epigenetics. We will then discuss evidence that ART and periconceptual maternal and paternal nutrition may lead to epigenetic alterations very early in pregnancy, and how each pregnancy experiences developmental programming based on signals received by and from the dam. Lastly, we will discuss current research on strategies designed to overcome or minimize the negative consequences or, conversely, to maximize the positive aspects of developmental programming.

## 1. Introduction—Developmental Programming

An important concept in developmental biology and medicine is that of “developmental programming,” often termed developmental origins of health and disease (DOHaD), which is the concept that “insults” during embryonic, fetal, and postnatal life can have long-lasting effects on the offspring. These effects result in a much greater risk of acquiring various so-called “non-communicable diseases,” or NCDs, as infants and adults. Such NCDs include abnormal growth and body composition, as well as behavioral, cardiovascular, endocrine, gastro-intestinal, immune system, metabolic, and reproductive dysfunction, the latter of which is a major socioeconomic issue because of its role in infertility [1,2,3,4,5,6] The initial observations that led to the concept of developmental programming were based on epidemiological studies in humans. These studies suggested not only that an adverse intrauterine environment may lead to a greater incidence of NCDs in the offspring as adults, but also that poor maternal nutrition was “… an obvious suspect [7,8].” The initial observations also noted that developmental programming was associated with “Discordance between placental and birth weights …”

Both assisted reproductive techniques (ART; e.g., embryo transfer [ET], in vitro fertilization [IVF], in vitro production of embryos [IVP], and cloning), and maternal nutritional status (e.g., over- or underfeeding or feeding specific nutrients) have profound effects on embryonic development during the periconceptual period (i.e., around the time of fertilization/conception) and continuing into early pregnancy, which is the period of embryonic/fetal organogenesis and placentation [1,5,6,9,10,11]. As discussed in several recent reviews [12,13,14], paternal nutritional status, via effects on sperm gene expression and epigenetic landscape, extracellular vesicles, and seminal plasma content, has been shown to contribute to embryo quality and affect embryonic/fetal and placental development in cattle [15,16], rodents [17,18,19,20], and sheep [21,22] models.

Importantly for the topic of this review, functional defects due to maternal under- or overnutrition or ART that have been documented during the periconceptual period include reduced embryonic development to blastocyst stage after IVF and embryo culture [23], reduced embryo–fetal growth [23,24], and altered nutrient supply to the developing fetus [25,26,27,28,29]. Assisted reproductive techniques also altered placental angiogenesis [9,30,31,32], global DNA methylation [24], and expression of steroid receptor mRNA and proteins [11,33,34,35]. Additionally, assisted reproductive techniques and moderate maternal nutrient restriction altered expression of gap junctions and chemokines and chemokine receptors in placenta [36,37]; altered expression of endogenous retroviruses, interferon-tau, and pregnancy-specific protein-B, as well as nutrient transporters, in placenta [25,27,38,39]; and altered gene expression during early development of fetal organs [40].

As recently reviewed [1,6,41,42,43], the effects of ART and nutritional status of the parents are probably mediated via “programming” of embryonic/fetal and placental gene expression, as reflected by changes in their epigenetic landscape [17,44]. Such epigenetic changes may underlie developmental programming of growth, development, and function of organs of the fetus later in pregnancy and the offspring postnatally. As discussed in the opening paragraph, they also potentially lead to long-term changes in organ structure and function in the offspring during infancy and continuing into adulthood, leading to behavioral, cardiovascular, immune system, metabolic, and reproductive dysfunction.

Some of the programming effects of ART and periconceptual parental nutrition likely depend on the epigenetic profile the oocyte or the sperm. The oocyte [10] and sperm [14,15,17,22,45,46,47,48,49,50,51] both contribute mRNAs, small non-coding RNAs, and proteins to the embryo, and these have profound influences on embryonic development before activation of the embryonic genome [10,52,53,54]. In addition, both the maternal (oocyte) and paternal (sperm) genomes can “transfer” their DNA methylation status to the embryo, and this is influenced by ART and nutritional status [10,15].

In this review, we will discuss the evidence that ART and periconceptual maternal and paternal nutritional status may lead to epigenetic alterations very early in pregnancy, ultimately determining events that lead to developmental programing, both during fetal life and postnatally. We also will discuss the effects of ART and maternal nutrition on reproductive function in the male offspring that may lead to transgenerational programming of development. Lastly, we will discuss current research strategies designed to overcome or minimize the negative consequences or, conversely, to maximize the positive aspects of developmental programming.

## 2. Evidence for Programming by Assisted Reproductive Techniques

Comparison of placental development in natural pregnancies and pregnancies achieved by various ART, such as after transfer of embryos created through cloning or IVF, has demonstrated numerous significant effects of ART on placental and fetal growth and development, as well as offspring outcomes in several species [9,11,34]. In various animals, including mice, cattle, and sheep, impaired placental steroid metabolism, abnormal offspring syndrome, increased duration of gestation, and altered placental vascular development, have been reported with the use of ART [9,11,23,24,32,33,34,35]. In addition, in sheep, use of ART significantly decreased fetal size at the third week of pregnancy and this was accompanied by decreased placental vascularity and cell proliferation, and altered expression of genes involved in epigenetic processes (e.g., 5-methyl cytosine and DNA methyl transferases), as well as factors involved in regulation of placental growth (e.g., steroid hormone receptors) and angiogenesis (e.g., several angiogenic and growth factors [24,32,33,35,55]). For early pregnancy in cows, both greater and lesser crown-rump length have been reported for fetuses created in vitro and then transferred compared to fetuses created in vivo [56,57].

In sheep, we, and others, have recently demonstrated that utero–placental vascularization, expression of several angiogenic factors, receptors for progesterone and estrogen, and DNA methyl transferases, as well as global DNA methylation and markers of growth all were altered in the developing placenta during early pregnancy after transfer of embryos obtained through IVF or in vitro activation (IVA; parthenotes, which have only a maternal genome [9,24,32,33,34,35,58]). One of the very interesting observations from this work is that even when they are derived from naturally mated ewes and transferred to recipients, the embryos show many of the same defects as IVP embryos [9,24,32,35], implying that improvements in embryo collection, culturing, and transfer methods are sorely needed.

These observations also suggest that changes in DNA methylation or histone modifications observed in early embryos of several species (cattle, mice, sheep) after ART [59,60,61,62,63] continue during the critical period of placentation in early pregnancy. In addition, based on observations later in pregnancy, we have suggested that these placental developmental defects probably contribute to poor placental vascularization and function later in pregnancy, ultimately leading to altered fetal growth and development and the poor pregnancy outcomes associated with ART [9,11,34].

Such epigenetic defects leading to altered placental development and gene transcription have been observed after IVF, but especially in clones, not only in sheep but also in cattle and mice [64]. These defects include altered transcription of imprinted genes, which are thought to be critical for normal placental development [65,66].

## 3. Evidence for Programming by Maternal and Paternal Nutrition

In adult women, extremes of pre-pregnancy body mass index (BMI), or, more commonly, BMI at the first prenatal exam, and both inadequate and excessive gestational weight gains are widely associated with a variety of negative obstetrical outcomes [42,67,68,69,70,71,72,73]. While few of these clinical studies have been able to cleanly study the separate and joint effects of initial BMI and gestational intake on pregnancy outcome, the broad consensus is that a very high pre-pregnancy BMI and/or high gestational weight gains are associated with an increased risk of preeclampsia, gestational diabetes, preterm and caesarian delivery, stillbirth, fetal macrosomia, and, in some cases, fetal growth restriction. In contrast, a low pre-pregnancy BMI and/or low gestational weight gains are commonly associated with an increased risk of preterm delivery and fetal growth restriction.

Despite these observations in adults, there is a paucity of similar data evaluating the effects of BMI and nutritional extremes on pregnancy outcome in pregnant adolescent girls. Nevertheless, there is some consensus that dietary intakes of pregnant adolescents are often lower than recommended in terms of energy and a range of micronutrients [71,74]. In addition, there also is evidence that pregnant adolescents, particularly those less than 15 years of age, are at risk of excessive gestational weight gain [75], similar in magnitude to the weight change measured in the overnourished and rapidly growing adolescent dams in one of our nutritional models of the pregnant adolescent ewe lamb [71]. Moreover, fetal growth restriction has been reported in mothers who are still growing or in mothers who have not achieved their adult height at the time of conception [76,77,78].

Maternal nutritional status during the periconceptual period and early pregnancy has profound effects on embryonic/fetal and placental development. For example, maternal under- or overnutrition during the 6 weeks preceding oocyte collection dramatically affect the rates of IVF and subsequent development of embryos to blastocyst stage in vitro [23,79]. In addition, underfed and overfed sheep had altered serum insulin, insulin-like growth factor-1, leptin, progesterone, and (or) estradiol concentrations, indicating they were affected metabolically as well [23,80]. Other studies reported decreased rates of fertilization with IVF and (or) decreased rates of early embryonic development in ewes or cows fed a low- (nutrient-restricted) or high- (ad libitum intake) energy diet during the periconceptual period [81,82,83,84,85]. Conversely, others have shown that an altered plane of nutrition during the periconceptual period had either positive or no effects on blastocyst formation and/or embryo health in sheep [83,86]. Interestingly, premating nutrition altered mRNA expression in oocytes and follicular cells, which may account for reductions in reproductive performance of ewes fed restricted diets [87]. Maternal undernutrition and folate supplementation during the periconceptual period also altered the methylome of sperm in the offspring [22]. In addition, maternal undernutrition reduced the proportion of embryos reaching the blastocyst stage after IVF, but folate supplementation reversed these effects (Table 1).

In cows, the effects of diet before and during the periconceptual period on oocyte quality measured by rates of fertilization with IVF and blastocyst formation, and cell number per blastocyst, depend on body condition (i.e., adiposity) and diet composition [88,89]. High levels of food intake improved postfertilization embryonic development for animals in low body condition, but reduced postfertilization embryonic development for animals in good body condition [87]. In addition, hyperinsulinemic cows with good body condition that were fed a high-energy diet produced fewer oocytes and had lower blastocyst yield after IVF [89]. Moreover, dairy cows that were fed a high-protein and high-energy diet exhibited altered endocrine and metabolic function and were frequently in negative energy balance, which is associated with low fertility, perhaps due to low-quality oocytes and embryos [90,91,92]. It also has been suggested that genetic factors also may contribute to diminished oocyte and embryo quality [91,92].

Maternal undernutrition during the periconceptual period in sheep results in altered fetal growth trajectory, altered fetal hypothalamic–pituitary–adrenal axis development, accelerated maturation of fetal adrenal glands, altered fetal pancreatic development, altered insulin signaling, and altered amino acid metabolism, all associated with an increased rate of premature birth and high rates of postnatal mortality [93,94,95,96]. How maternal underfeeding during the periconceptual period affects offspring growth and development is unknown. Overall, the available data indicate that the maternal diet during the periconceptual period alters the epigenetic status of the oocyte or early embryo [97], leading to short-term (e.g., effects on oocyte quality and thus the rates of fertilization and early embryonic development) and/or long-term (e.g., effects on offspring development and timing of birth, and thus developmental programming) effects. Recently, Sinclair and Singh [97] suggested that specific maternal dietary components such as vitamin B12 and methionine fed to ewes during the periconceptual period affect DNA methylation, insulin resistance, and blood pressure in the offspring.

Data from human and rodent models clearly show that developmental programming effects arise from paternal contributions. Authors reviewing 45 publications reporting data from rodent models presented overwhelming evidence that developmental programming extends to the F2 generation and beyond, with several of the papers reviewed presenting evidence of a paternal contribution to developmental programming [98]. Paternal diet has been shown to affect growth, metabolism, and reproductive traits in the offspring of rodents and sheep, potentially across multiple generations [12,14,17,18,19,20,21]. Messages carried in a father’s semen after exposure to alcohol, drugs, or an obese state result in altered development of their children [99,100]. The impacts of sire obesity can be long lasting, with effects on post-pubertal semen development in male offspring and the negative effects further exacerbated when offspring also received a high-fat diet [101].

At least some of the programming effects of paternal nutrition may involve epigenetic alterations in the father’s sperm [15,16,21,102]. Small non-coding RNAs are implicated in paternal programming by targeting maternal RNAs, or by entering the nucleus to directly alter transcriptional programming of the embryo [103]. Injecting purified sperm RNAs from male mice exposed to high-fat diets into normal zygotes resulted in offspring with impaired glucose tolerance and elevated blood glucose and insulin levels [50]. Injection of sperm microRNAs into wildtype oocytes resulted in metabolic and behavioral changes in the offspring [104]. When microRNA from stressed males was injected into fertilized oocytes of control females, emotional response and microRNA in the hippocampus (the emotional control center of the brain) were altered in the offspring [105].

In rodent models, fat and protein content of paternal diets have altered gene expression and function of key metabolic tissues in the offspring (liver and muscle; [12,20]) and feeding elevated concentrations of methyl donors (Choline, Betaine, Vit B6, Folate, and Vit B12) to boars resulted in offspring with differential methylation patterns in liver and muscle and altered body composition (reduced backfat and greater percentage of shoulder) [106]. Targeted feeding of rumen-protected methionine to rams resulted in changes in sperm methylation and was associated with altered weight at puberty and scrotal circumference of the offspring [21]. Diet of bulls during the early postpubertal period (1 year of age) affected sperm characteristics and concentrations of minerals and metabolites in seminal plasma [107,108], and a model of divergent nutrition in mature bulls providing either global nutrient restriction or excess resulted in differential expression of 769 transcripts in their sperm, including genes related to de novo methylation and histone to protamine transition [109].

## 4. Strategies to Improve Embryo Development and Pregnancy Outcomes

### 4.1. Reproductive Fluids

Coy and colleagues have shown that oviductal fluid, as well as specific proteins secreted by the oviduct, improve the rates of fertilization and embryonic development in vitro and in vivo, resulting in greater pregnancy rates and improved pregnancy outcomes with ART [110,111,112,113]. This technology holds great potential to overcome poor fertility and also to maximize the impact of elite genetic stock, and thus to dramatically improve the efficiency of livestock production. Whether maternal diet affects oviductal gene or protein expression, and whether such effects influence the ability of oviductal secretions to improve fertilization, embryonic development, or pregnancy outcomes has received little attention. However, we recently showed that restricted dietary intake does not affect protein expression of a major protein secreted by the oviduct in beef cows, namely oviductal glycoprotein 1 (OVGP1; [114]).

### 4.2. Supplementation

Targeted dietary supplementation of nutritionally compromised parents also is an important strategy being pursued by several groups of investigators. For example, Bazer et al. [115] have suggested that dietary supplementation of specific dietary amino acids that are present in uterine secretions (histotroph) could improve embryonic development. Such amino acids include serine and methionine, both of which are involved in one-carbon metabolism, as well as arginine, lysine, and histidine, which are targets of methylation when incorporated into histones. Arginine also improves feto-placental development in rodents [116,117,118], sheep [119,120,121,122,123], swine [124,125,126,127], and humans [128,129,130], potentially due to its involvement in generation of nitric oxide and polyamines [115,131].

The placenta transports amino acids from the maternal circulation and histotroph across the trophoblast/chorion via amino acid transporters ([115]. The cationic amino acid transporters (SLC7A1, A2, and A3) are expressed in uterine luminal and glandular epithelia and also in trophoblasts, and expression of two of these is stimulated by treatment of ewes with progesterone (SLC7A1 and A2) or, in the case of SLC7A2, by administration of interferon-tau (a conceptus product [132]). Expression of SLC1A5, a neutral amino acid transporter, in uterine luminal and glandular epithelia also is stimulated by both progesterone and interferon-tau treatment [133]. Progesterone treatment also increases the concentrations of fructose in uterine luminal fluid (histotroph; [134]).

**Figure 1:**
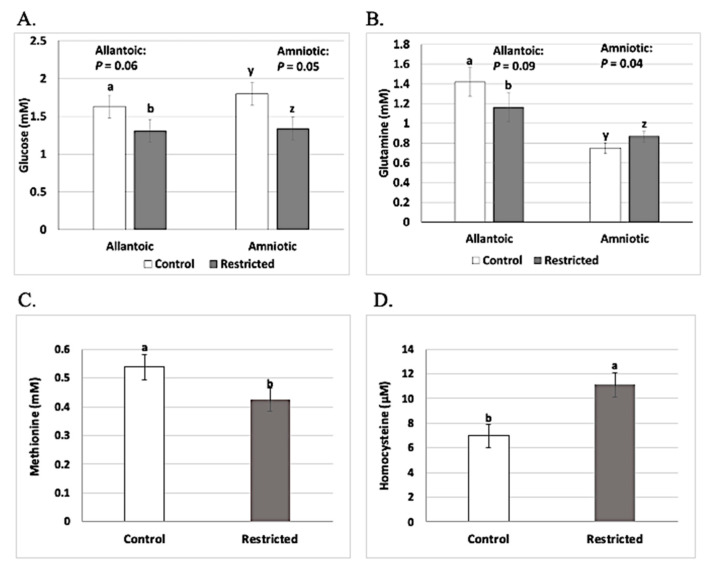
Comparison of (**A**) glucose concentrations in allantoic and amniotic fluid, (**B**) glutamine concentrations in allantoic and amniotic fluid, (**C**) methionine concentrations in allantoic fluid, and (**D**) homocysteine concentrations in maternal serum of heifers receiving control or restricted dietary treatment from the day of mating (d 0) until d 50 of gestation. Treatments provided for 0.5 kg of gain/hd daily vs. −0.08 kg of gain/hd daily between d 0 and d 50 of gestation for control vs. restricted heifers, respectively. Figure from Caton et al. [1]. a,b and y,z for (**A**,**B**) bars significantly different, with *p*-value as indicated; a,b for (**C**,**D**), bars differ significantly (*p* < 0.10).

Recently, our research group has shown not only that hexose and amino acid transporters are expressed in bovine utero-placental tissues, but also that expression of several of these transporters is influenced by maternal dietary restriction during early pregnancy (days 16 to 50 after mating [25,27,135,136,137]). In addition, maternal dietary intake affects the concentrations of hexoses (glucose and fructose) and several amino acids in allantoic and amniotic fluids, as well as homocysteine in maternal blood (Figure 1 [1]), all of which imply a profound effect of maternal dietary intake on maternal and embryo/fetal metabolism, including 1-carbon metabolism.

Another promising strategy is dietary supplementation with vitamins and minerals. In a recent series of studies, we have shown that providing a dietary vitamin–mineral supplements to beef heifers before mating and continuing for the first trimester of pregnancy affected maternal hormonal and mineral status, fetal organ weight, concentrations of amino acids in fetal fluids, abundance of fat soluble vitamins in fetal liver, placental nutrient transporter expression, and placental gene expression [28,29,135,136,137,138,139,140,141].

Targeted nutritional supplementation of one-carbon micronutrients to either the parents or cultured embryos is another strategy to “rescue” compromised embryos. This approach is based on in vivo and in vitro studies and the fact that the early embryo undergoes dramatic changes in methylation of the majority of its genes [17,142,143,144,145,146,147,148,149]. For example, in zebrafish, a parental diet deficient in one-carbon micronutrients altered oocyte production and one-carbon metabolism in the parents, as well as embryonic gene expression of pathways involved in the one-carbon cycle and lipid transport [150]. In dairy cows, maternal methionine supplementation caused significant changes in the transcriptome of the preimplantation embryos [151]. Recently, we have shown that supplementation of one-carbon metabolites during culture of bovine embryonic tracheal fibroblasts improves their mitochondrial respiration and growth rates and alters their DNA methylation status [26].

However, dietary supplementation of 1-carbon metabolites may also have detrimental effects [17,19]. For example, male mice fed folate-deficient diets had altered DNA methylation in their sperm; in addition, their offspring exhibited differential gene expression in their placentas as well as increased incidence of birth defects, including craniofacial and musculoskeletal malformations [17].

## 5. Conclusions and Future Directions

It seems clear that both ART and parental nutritional status, and probably a host of other “stressors” as well (e.g., parental age; environmental conditions, including temperature/humidity, elevation, and contaminants; relational or economic stress, etc.) can affect developmental competence of gametes and embryos [3,4,5,6,9,11,42,43,152]. It also seems clear that the periconceptual period is particularly important in terms of programming of embryonic and feto-placental development [1,85,93,95,153]. Thus, research on factors that affect gametes, including those during IVP of embryos, such as reproductive fluids or culture media supplements, should receive much more emphasis in the future. In addition, research to improve embryo culture, storage, and transfer methods should also be an area of emphasis.

Recent models by our group have gone beyond the extremes of dietary restriction and excess, and focused on a range of experimental conditions that are normally found in production scenarios. Differences in offspring outcomes, nutrients found in fetal tissues and fluids, and gene expression in placenta and fetal organs were observed in these models of moderate dietary alterations in maternal nutrient supply [28,29,137,138,139,140,141], which indicates to us that developmental programming may be present in a much larger proportion of pregnancies than given credence to in the literature.

We, therefore, propose that there is a continuum of developmental programming and that each pregnancy is uniquely programmed; with alterations in epigenetic signals generated in response to dietary environmental factors, stress, and other signals experienced by the dam. Related to this, an area that should receive much more attention in the future is management and therapeutic strategies to take advantage of the positive effects of developmental programming. This idea is based on the suggestion that developmental programming ultimately is adaptive and should improve the fitness of the individual [154,155]. In keeping with this focus, our studies of maternal nutritional status during the first 50 days of pregnancy in cattle have shown that the majority of genes in the fetal liver, muscles, and the brain were upregulated in fetuses from nutrient-restricted dams. We, therefore, suggested that upregulation of genes may represent an adaptive, rather than a negative, response to maternal nutrient restriction [40]. Consistent with this observation, cattle and other ruminants (deer, goats, sheep, etc.) that graze rangelands or other “extensive,” pasture-based systems are often nutrient restricted during early pregnancy [156,157,158,159]. Thus, they are well adapted to yearly cycles of low forage quality and availability and, therefore, to severe, short-term fluctuations in body weight and condition.

This idea of adaptive versus maladaptive responses to maternal malnutrition also has relevance to humans, as low caloric intake and malnutrition, usually associated with poverty, are experienced by many pregnant women worldwide, with severe consequences for pregnancy outcomes. Such consequences include low and very low birth weights, high rates of postnatal morbidity and mortality, and long-term consequences for health and productivity of the offspring, including stunting and wasting [67,160,161,162,163,164]. Thus, a much better understanding is needed of whether changes in expression of fetal and placental genes are adaptive, and therefore beneficial, or maladaptive, and therefore detrimental, to the offspring in both the short and long term.

Lastly, research on potential solutions to the negative consequences associated with programming of embryonic development has shown great promise. For example, the recent studies in which media were supplemented with oviductal or uterine luminal fluids have shown improvements in survival and development of embryos produced in vitro. Similarly, providing targeted dietary supplements to nutritionally compromised parents has shown improved developmental outcomes of offspring, including their reproductive function, thereby impacting subsequent generations. More work should be done, however, on supplementation of embryo culture media with various nutrients and hormones, and supplementing nutrients that provide substrates for key epigenetic processes. Due to their potential long-term benefits across generations and across species, we believe these efforts should continue and even be expanded.

## Figures and Tables

**Table 1:** Outcomes of in vitro fertilization (IVF) using sperm from lambs born to Control, Undernourished or Folic acid-Supplemented ewes.

Nutritional Treatment	Oocytes (n)	2-Cell Stage Embryos (%)	Blastocysts (%)
Control	178	56/178 (31.5)	26/178 (14.6)
Undernourished	186	44/186 (23.6) ^b^	10/186 (5.4) ^a,b^
Folic acid-supplemented	118	46/118 (39)	16/118 (13.5)

^a^*p* < 0.005 between Undernourished and Control; ^b^ *p* < 0.05 between Undernourished and Folic acid-supplemented; Fisher’s exact test. Table from [22].

## Data Availability

Not applicable.
